# “We Don’t Assume That Everyone Has the Same Idea About Health, Do We?” Explorative Study of Citizens’ Perceptions of Health and Participation to Improve Their Health in a Low Socioeconomic City District

**DOI:** 10.3390/ijerph17144958

**Published:** 2020-07-09

**Authors:** Marja A. J. G. de Jong, Annemarie Wagemakers, Maria A. Koelen

**Affiliations:** 1 GGD IJsselland, Zeven Alleetjes 1, 8011 CV Zwolle, The Netherlands; 2 Health and Society, Social Sciences Group, Wageningen University & Research, P.O. Box 8130, 6700 EW Wageningen, The Netherlands; annemarie.wagemakers@wur.nl (A.W.); koelen@caiway.nl (M.A.K.)

**Keywords:** citizen participation, health promotion, perceptions on health, concept mapping, health inequities

## Abstract

In community health promotion programs that aim to reduce health inequities, citizen participation is recommended, as it strengthens citizens’ active involvement and has a positive impact on health. A prerequisite for citizen participation is recognizing and incorporating citizens’ perceptions of health. Therefore, this study aimed to explore these perceptions and actions needed to improve the health of citizens living in a low socioeconomic city district. Concept mapping was used to actively engage community members as part of the action research method. Eleven community groups (n = 89 citizens) together with community workers participated in the study. Participants in all groups agreed that health entails more than the absence of disease, and therefore it is a multidimensional concept. Social relations, physical activity, positive life attitude, healthy eating, and being in control were important perceptions about health. Although the participants were aware of the relation between lifestyle and health, actions to improve health included doing things together, collaboration, self-confidence, focusing on possibilities, and socially shared meanings. Creating a supportive environment to address health behavior appeared to be the most important action for citizens to facilitate behavior change. Concept mapping helped to involve citizens and provided community workers with valuable information to shape the program together with citizens.

## 1. Introduction

In this study, the health perceptions of citizens in a socioeconomically deprived city district in the Netherlands were explored and subsequently used to develop a community health promotion program that aimed to reduce health disadvantages [1]. Socioeconomic health inequities persist in the Netherlands, with a 7-year difference in life expectancy between people with low and high socioeconomic status (SES), and an approximate 18-year difference in years lived in good perceived health [2]. It is uncertain how these differences will develop in the near future. We know that health inequities are a complex problem caused by the interplay between individuals; groups; communities; and multiple factors in the social, physical, and economic environment [3,4,5,6]. Therefore, strategies to reduce health inequities should be based on an ecological perspective, target the social determinants of health, and address factors at multiple levels and the interaction between factors [7,8,9]. Research on health inequities within different lifestyle groups has shown that individual health-related behaviors are bound up in activities that correspond to the person’s context (habitus) combined with his/her position in social space and subjective perceptions [10]. Consequently, health promotion ought not to target the individual health-related behavior or social participation/engagement in the neighborhood but rather consider the underlying drivers and their causes [11,12,13].

To date, health promotion programs have not been successful in substantially reducing the health gap between citizens with a higher and a lower SES [14]. It is therefore a challenge to develop more effective strategies to reduce this gap [6,15,16,17,18]. Health promotion, focusing primarily on lifestyle and risky behaviors, is an inadequate strategy for addressing social inequities in health [12]. Whereas citizens experience health as an integral part of everyday life, health promotion interventions often address isolated health themes or lifestyle factors, thereby focusing on the individual level [19]. Therefore, multi-level strategies are recommended in which community participation is made central, because that in itself has a positive impact on health [20,21]. Community participation—citizens’ active involvement and responsibility for activities—strengthens health literacy and empowerment [22,23,24]. In addition, the inclusion of members of vulnerable populations in the articulation of the problem and the development of the program is necessary because this takes into account the context of people’s lives [25,26] and empowers citizens to address health in a positive way, focusing on assets and resources [27].

A prerequisite for citizen participation is to recognize how citizens perceive health and the issues that are important for them regarding health and wellbeing. Studies that explored low SES citizens’ perceptions of health found that how people experience and define health differs depending on the context and the situation [28,29]. In general, citizens with a low SES are less likely to perceive the need for lifestyle advice and participate less often in lifestyle programs compared to citizens with a high SES [30,31,32]. A possible explanation is that these programs do not take sufficiently into account the low SES groups’ perspectives on health, life, and wellbeing. Furthermore, there appears to be a discrepancy in health perceptions between citizens and health professionals [33]. Knowledge about differences in perceptions, often gleaned through questionnaires, is used by health promotion professionals to develop new interventions or to adapt existing interventions [34,35]. Consequently, programs are often expert-driven and people do not recognize themselves, their concerns, or their problems and therefore do not see any reason to participate. Citizens do not make an active contribution to content and development, nor is their context taken into account. It is therefore recommended to be aware of these differences and to actively include citizens’ perceptions and citizen participation in health promotion activities and practice [36,37].

Therefore, the aim of the study was to explore the health perceptions of citizens in a low socioeconomic city district, together with an assessment of citizens’ needs and wishes. It is the first step in the development of a community health promotion program in the same city district in which citizens’ active involvement in program activities is put in the center. This means that the program’s activities are not chosen or planned beforehand but rather developed and implemented jointly by professionals and citizens, in their context, and based on the health perceptions and needs expressed by the citizens themselves. Consequently, the following research questions are formulated: What are the perceptions about health of citizens living in a low socioeconomic city district? What factors, in the perception of citizens with a low SES, contribute to their health and what actions do they need to improve their health?

## 2. Materials and Methods

### 2.1. Study Setting

This study is part of a larger study in which a community health promotion program—called Voorstad on the Move (VoM)—was developed, facilitated, and evaluated [1]. In line with national and local health policy [38], the aim of the program was to contribute to the improvement of health and to find ways to reduce health inequities [1,39]. VoM was implemented between July 2016 and January 2020 in a city district of 10,750 inhabitants in a town in the east of the Netherlands. In this city district, both the SES and the health status of inhabitants are relatively low compared with other parts of the city [40]. The VoM program puts three action principles at its center: citizen participation, intersectoral collaboration, and a health supportive environment [39]. At the start of the VoM program in 2016, a local team was formed consisting of community welfare workers, a neighborhood sports officer, and a health broker [41,42]. There appeared to be a strong and lively social infrastructure in this city district, and that was used by the health broker to join and build a network of existing community groups, healthcare and social workers, and volunteers. This was an essential part of this program’s bottom-up approach, where the community workers act to support the citizens in the identification of issues that are important and relevant to their lives, and this knowledge enables them to develop strategies jointly with citizens to resolve these issues [43]. Action research was integrated in the program’s bottom-up approach as a strategy for both facilitation and evaluation purposes. The value of action research is that it reflects the core principles of health promotion, such as participation and empowerment [15,44,45,46,47,48].

### 2.2. Study Design

To explore health perceptions in this study, the concept mapping (CM) methodology was used. CM refers to any method or structured process used to produce a picture or map of the ideas or concepts of an individual or group about a complex multidimensional problem [49,50,51]. The CM methodology is well-suited to actively and directly engage community members, as one of CM’s major strengths is the inclusion of participants in the interpretation and analyses of maps constructed by the mapping groups [52]. All the steps (1–6) of the CM process developed by Trochim [53] were followed, together with the participants in the groups. Because of the action-oriented character of this study and the importance of citizens’ involvement, all the steps were conducted with citizens accompanied community workers. This is different from other studies, in which steps 5 and 6 are conducted by professionals [54,55].

### 2.3. Participants: Recruitment and Response

From April to November 2017, community workers guided the recruitment of existing community groups and accompanied them in the group sessions. The groups, which were active in community centers, elementary schools, and residents’ associations, were asked to participate in two group sessions each. Every effort was made to include a wide range of citizens involved in a variety of activities (e.g., physical activity or hobby), age, sex, and ethnic background. Eleven of the 14 groups that were invited agreed to participate. Three groups refused because of a lack of interest. Four groups participated only in the first session. The reasons for not participating in a second session varied from difficulty planning (n = 2), a strong variation in group composition per meeting (n = 1), and lack of motivation to attend a second session (n = 1). In two groups, both sessions were held during one meeting. A total of 89 citizens participated in this study, all inhabitants of Voorstad city district, varying from 4 to 11 participants per group (Table 1). Groups 1–5 can be characterized as activity groups—e.g., yoga or walking—6–8 are residents’ groups that gather for social interaction, and groups 9–11 consist of volunteers who work together on a specific mission—e.g., running a community center or a play garden.

### 2.4. Ethical Approval

The project proposal was reviewed and approved by the Social Sciences Ethics Committee (SEC) of Wageningen University and Research. The Committee concluded that the proposal deals with ethical issues in a satisfactory way and that it complies with the Netherlands Code of Conduct for Research Integrity (*Date 18-10-2018*). The participants were recruited on a voluntary basis, could withdraw at any point, and were fully informed about the research activities. Oral consent was obtained from all the participants.

### 2.5. Procedure: Concept Mapping

The community health promotion program started by becoming acquainted with the existing community groups by inviting them for group interviews about health. This served two mutually reinforcing goals: (1) to gain insight into citizens’ perceptions about health and important health issues, and (2) to encourage citizens to develop or (continue to) participate in activities that contribute to their health and wellbeing.

The group sessions took place at the meeting points of the community groups (e.g., community center), because the participants were familiar with these places. The sessions were facilitated by trained and experienced moderators. All the moderators received the same instructions and scripts. The research assistant supported, observed, and took notes during the group sessions. In addition, the health broker or another community worker was present, because they were responsible for contact with the community groups. These professionals did not participate in the discussion but did support the actions that emerged from the discussions.

The CM process developed by Trochim [53] follows a six-step process of (1) preparation, (2) idea generation, (3) structuring, (4) representation, (5) interpretation, and (6) utilization [53,56]. Two group sessions were held with each community group (Table 2). Steps 2–4 were covered in the first session. The second group session consisted of steps 5 and 6. The second session was planned within two weeks of the first session. Sessions lasted on average 55–60 min.

• Step 1. Preparation step: identification of the focus for the mapping project, selection of participants, and determination of the project schedule and logistics:

The first step is described in detail in the recruitment and procedure sections.

**Figure 1:**
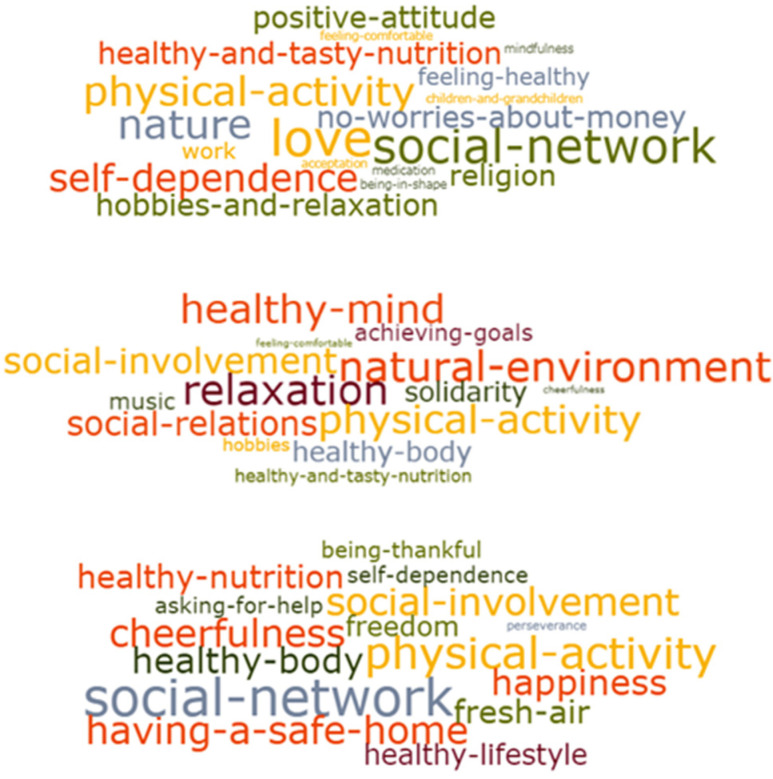
Word clouds of three different groups.

• Step 2. Generation of ideas through brainstorming by engaged community members:

In the first session, the question “What does feeling healthy mean to you?” was discussed. The moderator asked the question to the group and provided extra explanation in case it needed, thereby stimulating the participants to think about positive words or statements. The participants individually wrote words or statements that they associated with health on separate cards. From all the words gathered, a so-called word cloud was composed using an online tool [57] (Figure 1).

• Step 3. Structuring the ideas through clustering:

The groups composed clusters by putting together the words or statements mentioned by the individual participants. To compose the clusters, the meaning of the words was extensively discussed, facilitated by the moderator. Each cluster was assigned a label that the group agreed upon. This resulted in a minimum of 6 clusters in one group to a maximum of 14 clusters in other groups.

• Step 4. Representation: individual priorities:

The participants were asked to prioritize the clusters in an individual top 3. The priorities taken together resulted in a ranking of the clusters from 1 (most important) to 10 (least important) for each group.

**Figure 2:**
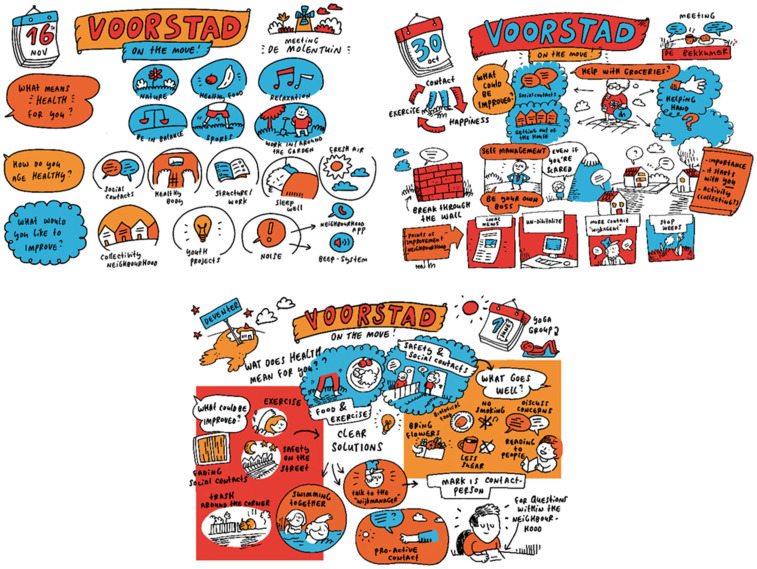
Visualization of the action plans of three different groups.

• Steps 5 and 6. Interpretation and utilization:

In steps 5 and 6, the participants were actively involved and took the lead in the interpretation and utilization of the results of steps 2 to 4. The word cloud from the first session was presented as a reminder. The needs and wishes for retaining and improving health were addressed by asking the following questions: “What is going well?” “What changes concerning your health would you make?” and “What do you need to retain or improve your health?”. The participants were challenged to explore specific actions and ideas. Resources, facilitators, and barriers relating to health-improving actions were explored. The results were again represented visually by a cartoonist and served as an action plan for each group (Figure 2). The cartoonist attended five groups to make the live report and used audio recordings from the other groups to visualize the results. All the participants received a hard copy of the picture. The results of all sessions were brought together and used as input for the health promotion programme.

### 2.6. Overall Data Analysis

The CM steps 1 to 6 were analyzed within the community groups, as described in the CM steps. In addition, the researcher made an overall ranking of the perceptions on health and analyzed comprehensively all the data gathered in the 11 groups to identify needs, barriers, and facilitators. The sessions were audiotape recorded and transcribed verbatim. The data from the sessions (e.g., cards, titles of clusters, and scoring) were collected, and field notes were compiled. A thematic content analysis approach was applied to the data (transcripts, results, and field notes), supported by ATLAS.ti 8.4 (ATLAS.ti Scientific Software Development GmbH, Berlin,). Two researchers performed this analysis, which involved open, axial, and selective coding. Firstly, the data from the first three focus groups were individually read, marked, and coded (open coding). The researchers discussed and compared the codes and reached a consensus on the use of codes. Next, the codes were individually categorized and clustered into themes (axial coding). Once the themes and interpretations had been discussed, a thematic map was developed. Constant comparison was made across and within cases. An overall ranking of the clusters was composed by counting the number of groups that mentioned the cluster together with the group ranking. The results of the overall analysis were reported and discussed in a special meeting with community workers and in regular meetings with the project team.

## 3. Results

### 3.1. Perceptions of Health

A wide range of statements emerged from the first group sessions, varying from 22 to 162 statements per group. These included more general aspects about the meaning of health (e.g., cheerfulness, relaxation, social network, self-dependence), as well as aspects that were either facilitators or barriers (e.g., physical activity, fresh air).

Overall, seven clusters or perceptions of health were ranked highest (Table 3). The social relations cluster, including friends and family, was ranked on top together with the physical activity cluster. Physical activity was perceived to be good for your health, but also a way to do something together with others (a social activity), to relax, to spend time outdoors, and to de-stress. A positive life attitude or mindset—also called cheerfulness or happiness—was the third most important cluster of perceptions. In eight groups, the healthy eating cluster was discussed extensively. The participants perceived healthy eating as an important health-related behavior, similar to physical activity. The fifth cluster—being in control and empowered—was listed in seven groups and related to being able to decide and react by oneself. The relaxation and mental rest cluster was indicated as the positive opposite of stress and having troubles—e.g., having a mind that was occupied. In six groups, the natural environment was prioritized as an important cluster for health.

### 3.2. Needs and Barriers to Improving Health

In the second group sessions, a large variety of needs, barriers, and specific actions were discussed (Table 4). Doing things together with others and having colleagues, friends, or family members around to support you were mentioned frequently as prerequisites to retain or improve health. Self-confidence or self-reliance, thinking in possibilities instead of barriers, acceptance, and talking about the situation or problem and subsequently asking for help were also often identified as assets that are supportive of good health. On the other hand, physical restrictions and disabilities and chronic diseases were viewed as barriers to good health. In the groups (groups, 3–6, 11) with elderly citizens in particular, chronic disease and physical impediments were topics of discussion but were not written on the cards. In the majority of the groups, financial barriers were a main topic, as this highly affects citizens’ resources, e.g., to buy healthy food for their children. In the multicultural groups, the participants mentioned language and cultural habits as barriers to communicating and interacting with others in the neighborhood. The participants exchanged their experiences and provided suggestions on how to deal with the limitations resulting from diseases and impediments and other barriers that they met.

### 3.3. Actions to Improve Health

In five groups, the participants were convinced that they were doing well and they did not feel the need to improve their health or engage in actions other than what they already did. They did not suggest actions to improve health for themselves and the group. They pointed out that the group served as a meeting place, where they spent meaningful time together, could talk about happy and sad things, and ask one another for help. Therefore, no suggestions for other activities came up. Rather, the focus was on the group activity. For example, the walking group talked only about walking, and there was a strong consensus on the opinion that walking is the solution for everything and walking was what they were already doing!

In the other groups, only a few new actions were suggested, but there were individuals who had ideas and wishes about health-promoting activities (Figure 2): for example, go swimming together, start a conversation with and visit new neighbors, and keep the street clean.

The community workers who attended the group sessions acquired a broader view of the citizens’ health perceptions and their needs and facilitators. Moreover, by attending the group sessions, they were motivated immediately to initiate and support the participants’ proposed actions by removing practical barriers or taking the first step towards action together. The following actions resulted right away from the CM sessions:

- The language group and the yoga group participants went swimming;
- Two people got a biking buddy;
- Some language group participants took guitar lessons;
- The resident group participants made appointments to meet more regularly;
- One group organized a high tea to meet (new) neighbors.

## 4. Discussion

As part of a community health promotion program, this study aimed to explore the health perceptions of citizens living in a low socioeconomic city district. In addition, citizens were asked what factors, in their perception, contribute to their health and what actions could improve their health. Exploring these questions, using CM in two group sessions, stakeholders (professionals as well as citizens) were involved and provided the information to engage the community and to further develop the health promotion program jointly.

### 4.1. Health is a Multidimensional Concept

The citizens with low SES in this study were well aware of the relation between health and behavior and they were also very clear about what was important for them. In all the groups, health was considered a multidimensional concept. Participants agreed that health entails more than the absence of disease. Although several citizens had a (chronic) disease, they viewed themselves as healthy as long as they were not limited in their daily functioning, as was also found in the study of Lopez et al. [58]. Throughout the groups, which differed in age, cultural background, and the activities undertaken together, there appeared to be broad agreement on the clusters of statements regarded as perceptions on health and the priorities given to these perceptions. In the different groups, a consistent pattern emerged between perceptions, needs, and actions. Our study revealed seven perceptions that were perceived to be most important: (1) social relations and interactions, (2) physical activity, (3) positive life attitude, (4) feeling in control, (5) healthy nutrition, (6) mental rest, and (7) the natural environment. This is in accordance with the findings in recent research on perceptions of health and lifestyle in other SES groups [33,59,60,61,62].

### 4.2. Perceptions on Health and the Social and Natural Environment as Important Assets

The participants ranked the social relations perception as the most important one for health and indicated that what they needed to improve their health was doing things together, collaboration, and giving and asking for help. The groups in which they participate, the volunteers’ work, and social gatherings already provided considerably for their needs. The group meetings can—in themselves—be regarded as health promoting.

A positive life attitude, feeling in control, and mental rest—perceptions with high rankings in all groups—can be considered as aspects of mental health and citizens’ attitude towards life. Accordingly, a focus on possibilities, self-confidence, and acceptance were mentioned as supportive of good health. This indicates the importance of paying attention to the subjective dimensions that determine health judgements and the way in which citizens cope with circumstances [28]. The participants asked for different social activities in the community centers—e.g., a “Looking for sense” course nearby, in their own neighborhood and free of charge.

These findings show that it is necessary to create a supportive social environment to facilitate behavior change and improve health [63,64]. This corresponds with the view on social determinants of health, which recognizes that health behavior is greatly influenced by people’s environmental, socioeconomic, and cultural settings [12].

According to the participants, a supportive environment also refers to the physical (natural) environment. In the majority of the groups, the natural environment was extensively discussed as an important perception in relation to good perceived health. This was expressed by phrases such as fresh air, lots of green in the living environment, and spending time outdoors. The natural environment was connected to an active lifestyle, performing physical activities, but even more to social activities and mental relaxation. Citizens considered the natural environment a notable health asset and therefore a resource to maintain and sustain health and wellbeing [27].

### 4.3. Citizens and Professionals Working Together to Build a Health Promotion Programme

The co-production by citizens and community workers in this study, with the citizens taking the lead, resulted in changes in professionals’ views of health promotion. In general, a professional’s view of health promotion focuses on health behavior or lifestyle, referred to as determinants of health [19,65]. In our study, two lifestyle aspects—physical activity and healthy nutrition—had high rankings in health perceptions, but citizens hardly mentioned health-related behaviors needed to improve their health. Although they were aware of the importance of lifestyle and behavior in relation to health, this was not prioritized in the actions mentioned, nor was it sufficient to build the health promotion program on. This corresponds to contemporary theories of social practice that suggest that “interventions to improve the health of populations and create positive social change will be better served by targeting social practices rather than the attitudes, behaviour and choices of individuals” [66]. In these theories, health and wellbeing are considered outcomes of a set of social practices, part of everyday life, and not only the result of a healthy lifestyle [10].

Witnessing the process and outcomes of the group discussions, the community workers adopted the citizens’ perceptions, priorities, and needs. Moreover, these views and needs increased the professionals’ awareness of citizens’ health perceptions and helped them to develop actions and engage other professionals working in other disciplines—e.g., social workers, the district manager, neighborhood sport connectors, general practitioners, and physiotherapists. The multidimensionality of health, as expressed by the participants, should be reflected in the development and implementation of health promotion programs. These findings support the idea that interventions should always take into account the target group’s social environment and perceptions and should involve the target group in their development [67,68,69]. Altogether, the changes in professionals’ knowledge and attitude, together with the knowledge and understanding of the social determinants of health in a community, can affect practical action to improve health equitably [70].

### 4.4. Methodological Considerations

The CM method contributed to a great extent to the outcomes of this study by providing insights into low SES citizens’ health perceptions and at the same time fostering stakeholder participation in the VoM program. This study laid the basis for the VoM program. As described, the program started with the formation of a local team consisting of community workers that had already worked for several years in the city district. Furthermore, the health broker was an inhabitant of Voorstad; she knew many people in person. The local team proved to be a good starting point for the program, because the team members were able to reach the different community groups and keep them involved during the entire program. The CM process appeared to be a good mechanism to initiate dialogue with the community and a way to stimulate critical thinking across stakeholders in the community, as also stated by Risisky et al. [49]. It created an opportunity for community engagement in the VoM program. The community groups that took part in the study remained involved over the term of the program by participating in activities and research interventions. The discussion and reflection helped citizens to express their view on what they needed to stay healthy and improve health. However, the lack of ethnic variability in most of the groups affects the representativeness. Most existing community groups in this city district have a homogeneous composition regarding ethnicity and the majority of these groups are Dutch. The local team did an effort to recruit groups with Turkish participants, but these groups were reluctant to participate because of the language barrier.

An important contribution deriving from our way of applying CM is that participants contribute directly to data analysis by taking part in the discussion and in the interpretation of findings. Unlike other qualitative methods such as in-depth interviews or focus group discussions, in which the data are collected and then analyzed later by the researcher, CM ensures that the results directly reflect participants’ thoughts and perceptions [52]. The downside is that the participants may have influenced one another—e.g., by calling out loudly the words they wrote on the cards—before everyone had written their own cards. On several occasions, the respondents started to confer immediately with one another on their thoughts and perceptions. The moderator of the group sessions had an important role. The VoM moderators were very experienced and made a big effort to give room to all the participants, thereby diminishing the potential confirmation bias. As it is challenging to manage the group dynamics and the focus of the moderator should be on the group discussion between participants, the splitting of the facilitator role and the researcher role is recommended [71], as was the case in our study. The use of visual recordings such as the word clouds and the pictures of the action plans strengthened the discussion and reflection and thereby contributed to outcomes such as actions [54]. The word clouds proved to be useful as inputs for the discussion about needs and barriers in the second group session. The researcher used a content analysis facilitated by Atlas-ti to process all the information gathered in the CM process [72]

In this study, we worked with existing community groups, and this had several advantages but also limitations. Firstly, the groups are part of the social infrastructure in the neighborhood and formed a relatively solid base on which to build the community health program. Secondly, these groups were easy to reach because they were on the radar of welfare and other community workers and therefore easy to start with. Group sessions could take place during regular meeting times and thus did not put much extra strain on the participants. Thirdly, the participants knew one another and felt secure talking about their own health and health problems [73]. The participants in several groups corrected one another and gave advice to others on how to deal with the barriers raised.

Although several different individual actions resulted right away from the CM sessions, the groups did not suggest new group activities because these citizens were convinced that they were doing well already. This can be regarded as a limitation of working with existing community groups in this study. The participants already had their social network and did activities together. Other ways of getting in contact with the harder-to-reach groups or individuals in this city district should be explored, for example through housing associations, youth healthcare, or employment projects. It should be taken into account that this will be time and energy consuming. A cost-benefit assessment is recommended [35].

## 5. Conclusions

The results of the CM sessions showed a wide range of perceptions of health and the requirements and possibilities to improve health. Although most participants in the existing community groups did not take up new health promotion activities, the study helped to involve citizens and community workers. The results were used to develop the VoM program together. It has become clear that the focus in the health promotion program should be on the social dimensions of health, offering citizens different possibilities for action on demand and adapted to their wishes. Activities should have a positive approach and should take place in the neighborhood free of charge, thereby fostering social relations and networks.

CM, as part of action research, proved to be a very useful method for gaining insights into low SES citizens’ health perceptions and at the same time fostering stakeholder participation in the program. The use of visual recordings in the CM procedure strengthened the process and the outcomes.

## Figures and Tables

**Table 1:** Characteristics of the community groups and participants.

	Name	Participants Total Session * 1 2			Sex	Mean Age	Ethnic Background	Occupational Status	Educational Status
1	Adolescents’ group AG	11	11	- **	Male: 5 Female: 6	17 (*14–30*)	Dutch: 11	Student: 10 Employed: 1	Low: 4 Medium: 5 High: 2
2	Language group LG	10	8	7	Male: 2 Female: 8	40 (*27–66*)	Dutch: 2 Turkish: 4 Syrian: 2 Other: 3	Employed: 1 Unemployed: 8 Retired: 1	Low: 6 Medium: 2 High: 2
3	Yoga group YG	8	7	5	Male: 0 Female: 8	71 (57–79)	Dutch: 8	Employed: 2 Unemployed: 1 Retired: 5	Low: 4 Medium: 1 High: 3
4	Knitting group KG	9	8	9	Male: 0 Female: 9	73 (53–92)	Dutch: 9	Employed: 3 Retired: 6	Low: 6 Medium: 1 High: 2
5	Walking group WG	7	7	4	Male: 4 Female: 3	69 (64–77)	Dutch: 7	Unemployed: 1 Retired: 6	Low: 4 Medium: 1 High: 2
6	Residents’ group A RA	7	6	4	Male: 1 Female: 6	61 (22–77)	Dutch: 3 Turkish: 3 Indonesia: 1	Employed: 1 Unemployed: 2 Retired: 4	Low: 6 Medium: 1
7	Residents’ group B RB	10	10	- **	Male: 3 Female: 7	72 (57–82)	Dutch: 10	Employed: 1 Unemployed: 1 Retired: 8	Low: 8 High: 2
8	Residents’ group M RM	8	4	8	Male: 5 Female: 3	47 (16–69)	Dutch: 8	Employed: 2 Unemployed: 2 Retired: 2 Student: 2	Low: 3 Medium: 3 High: 2
9	Volunteers’ community centre VD	4	4	- **	Male: 3 Female: 1	69 (66–71)	Dutch: 4	Retired: 4	Medium: 2 Unknown: 2
10	Volunteers’ play garden VS	6	6	6	Female: 6	37 (31–47)	Dutch: 5 East Europe: 1	Unemployed: 6	Low: 6
11	Women’s group VC	9	9	- **	Male: 1 Female: 8	67 (44–87)	Dutch: 8 Polish: 1	Employed: 4 Unemployed: 1 Retired: 4	Low: 8 High: 1
	**TOTAL**	**89**			**Male: 24** **Female: 65**			**Employed: 15** **Unemployed: 22** **Retired: 40** **Student: 10**	**Low: 55** **Medium: 16** **High: 16** **Unknown: 2**

* The numbers of participants differed between the first and second focus group sessions; ** Group did not participate in the second focus group session.

**Table 2:** Short description of concept mapping procedure based on Trochim [52,53].

	1. Preparation	Recruiting participants and defining questions and focus of group sessions.
First group session ‘What does feeling healthy mean to you?’	2. Idea generation	Participants individually wrote words or statements that they associated with health on separate cards.
	3. Structuring the ideas	With all cards collected, the group composed clusters of words/statements that belonged together and assigned a name to each cluster.
	4. Representation	Participants individually selected the three most important clusters. The rankings resulted in a group rating from 1 (most important) to 10 (least important).
Second group session ‘What do you need to retain/improve health?’	5. Interpretation	The results of the first focus group session, clusters as well as ranking, were fed back in a second session. Needs and wishes for improving health were inventoried and discussed.
	6. Utilization	Resources, facilitators, barriers, and ideas about health-improving actions were explored. A visual representation of the results of this session was made by a cartoonist. All the results of both sessions were brought together and used as inputs for the VoM health promotion program.

**Table 3:** Clusters of statements/perceptions of health in order of importance.

Perceptions	# Number of Groups Mentioned	Quotes	Actions to Improve Health
Social relations	10	“Relations, I think, are very important, with other people. Has to do with health as well.” (WG) “Look, as soon as one doesn’t have social relations, you are getting lonely and loneliness is bad for your health.” (VD)	Participate in one of the community or activity groups Activities in neighborhood centers
Physical activity	10	“If you keep on moving, you experience; I feel healthy.” (YG) “When I’ve been swimming; I feel relaxed and then afterwards I can pay attention to my child and be fully present.” (VS)	Swimming lessons Biking buddy Walking, yoga, Zumba
Positive life attitude	9	“Just always putting the focus on positive things.” (AG) “Seize the day, that’s what I always say.” (YG) “To stay healthy, you need to think positively about all problems.” (LG)	No specific actions
Healthy eating	8	“Food and eating have different aspects, like enjoying it, but also you simply need it.” (RB) “Healthy eating, making tasty soup and … don’t eat too much.” (LG)	Cooking workshops (adolescents, Turkish women)
Being in control/empowered	7	“That I can decide about what to do and what not.” (YG) “Being able to do everything by yourself; self-dependence.” (RA)	Course “Looking for sense”
Relaxation/mental rest	7	“It’s a way of relaxing and taking time for myself.” (RM) “No duties, everything is allowed, well... everything …. Ha ha.” (LG)	No specific actions
Natural environment	6	“Spending time outside is relaxing, a kind of rest.” (YG) “Being outdoors is a piece of happiness.” (YG) “Fresh air also has something to do with it, with health.” (RB)	No specific actions

**Table 4:** Needs and barriers to improving health.

Supportive of Health	Barriers to Improving Health
Social environment, friends “….. because you are in contact, you matter again.” (YG) Doing things together, collaboration, giving and asking for help “… but there are people surrounding you, that care and want to give help.” (RB) “If you get started together, I mean having social interactions with other people, then it becomes easier to accept yourself as well.” (RM) Acceptance, openness about the situation “It does not work, or it works with some extra effort. That doesn’t matter. It’s all part of getting older, I always say.” (RB) Self-confidence, focus on possibilities “It is just that you should better not complain but just hop on your bike and go.” (KG) Character traits like perseverance, courage, being strong, and taking the initiative “With a strong character, one does everything with perseverance and confidence. A strong character is what you need.” (VS) A dog (pat) “With a dog, you get enough physical activity.” (KG) Bike, e-bike “A special low step through bike; very nice and now I can use it more.” (KG)	Cultural aspects “It depends on, I think, the family and culture you grow up with. What the habits are.” (KG) Beliefs, convictions “Your own thoughts can hinder you, you know.” (RB) Physical impediments “I have a lot of physical impediments. Still, I would feel like being the same as before; a very competitive person I am. And because of that, I’ve lost my quality of life.” (RM) “I really find it difficult not to be able to open a jar of marmalade for example.” (RB) (Chronic) Diseases and illness “I only have 50% lung capacity, so I am permanently, when doing something, I am always out of breath.” (RM) Financial aspects, money “A lot of things just cost a lot, for me too. I have four children and I am getting older and it all becomes very expensive.” (VS) Language “Often, things go wrong because of the talking and the language barrier that one has.” (RA)
